# A Review of Structural and Biomechanical Changes in the Cornea in Aging, Disease, and Photochemical Crosslinking

**DOI:** 10.3389/fbioe.2019.00066

**Published:** 2019-03-29

**Authors:** Brecken J. Blackburn, Michael W. Jenkins, Andrew M. Rollins, William J. Dupps

**Affiliations:** ^1^Cole Eye Institute, Cleveland Clinic, Cleveland, OH, United States; ^2^Department of Biomedical Engineering, Lerner Research Institute, Cleveland Clinic, Cleveland, OH, United States; ^3^Department of Ophthalmology, Cleveland Clinic Lerner College of Medicine of CWRU, Cleveland, OH, United States; ^4^Department of Biomedical Engineering, Case Western Reserve University, Cleveland, OH, United States; ^5^Department of Pediatrics, Case Western Reserve University, Cleveland, OH, United States

**Keywords:** cornea biomechanical properties, cornea, aging, crosslinking, crosslinking (CXL) corneal collagen, keratoconus

## Abstract

The study of corneal biomechanics is motivated by the tight relationship between biomechanical properties and visual function within the ocular system. For instance, variation in collagen fibril alignment and non-enzymatic crosslinks rank high among structural factors which give rise to the cornea's particular shape and ability to properly focus light. Gradation in these and other factors engender biomechanical changes which can be quantified by a wide variety of techniques. This review summarizes what is known about both the changes in corneal structure and associated changes in corneal biomechanical properties in aging, keratoconic, and photochemically crosslinked corneas. In addition, methods for measuring corneal biomechanics are discussed and the topics are related to both clinical studies and biomechanical modeling simulations.

## Introduction

The visual system is a beautiful case study of biological relationships between structure, mechanics and function. To sharply focus an image onto the retina, the eye requires a tightly toleranced tissue structure. Providing around two-thirds of the eye's focusing power (Sridhar, 2018), the cornea is the primary refracting component of the human visual system. Micrometer-scale changes in the geometry of the anterior corneal surface can produce visually significant changes in refractive function on the order of several diopters. While this relationship can be leveraged intentionally in refractive surgical procedures to reduce the need for optical correction in the form of glasses or contact lenses, less desirable changes in corneal optical performance can arise from inter-individual variation or pathology of the cornea's microstructure and constitutive mechanical properties since these factors directly influence corneal shape and stability.

Understanding these relationships affords an important opportunity to improve clinical practices in diagnostics and treatment. For instance, there is evidence to suggest that in some degenerative diseases, the cornea exhibits mechanical changes before the commonly-used shape metrics indicate a pathologic state (Roy et al., 2013). Additionally, there is evidence of important age-dependent differences in corneal response to radial keratotomy, astigmatic keratotomy, and laser *in situ* keratomileusis (LASIK) that lead to systematic overcorrection of older patients or undercorrection of younger patients (Waring et al., 1987; Akura et al., 2000; Roudakova et al., 2000) and higher risk of corneal destabilization after refractive surgery in younger patients (Randleman et al., 2008a). This review will summarize what is known about the constitutive and biomechanics of the cornea in healthy, aged, diseased, and biomechanically altered states. In examining this knowledge base, it is also crucial to discuss the differences between various methods of measurement as well as how these results may be related back to clinical practice.

## Structural and Constitutive Anatomy of Cornea

The cornea contains intricate, multi-scale structures with distinct patterns of fiber organization in different layers. In addition, the composition of each layer is distinct, with variations in the type and density of collagens, elastins, fibronectins, laminins, and proteoglycans. These different organizational and constitutive motifs give rise to widely differing mechanical properties. This section will briefly summarize the major organization and constitution of each layer of the cornea.

**Figure 1:**
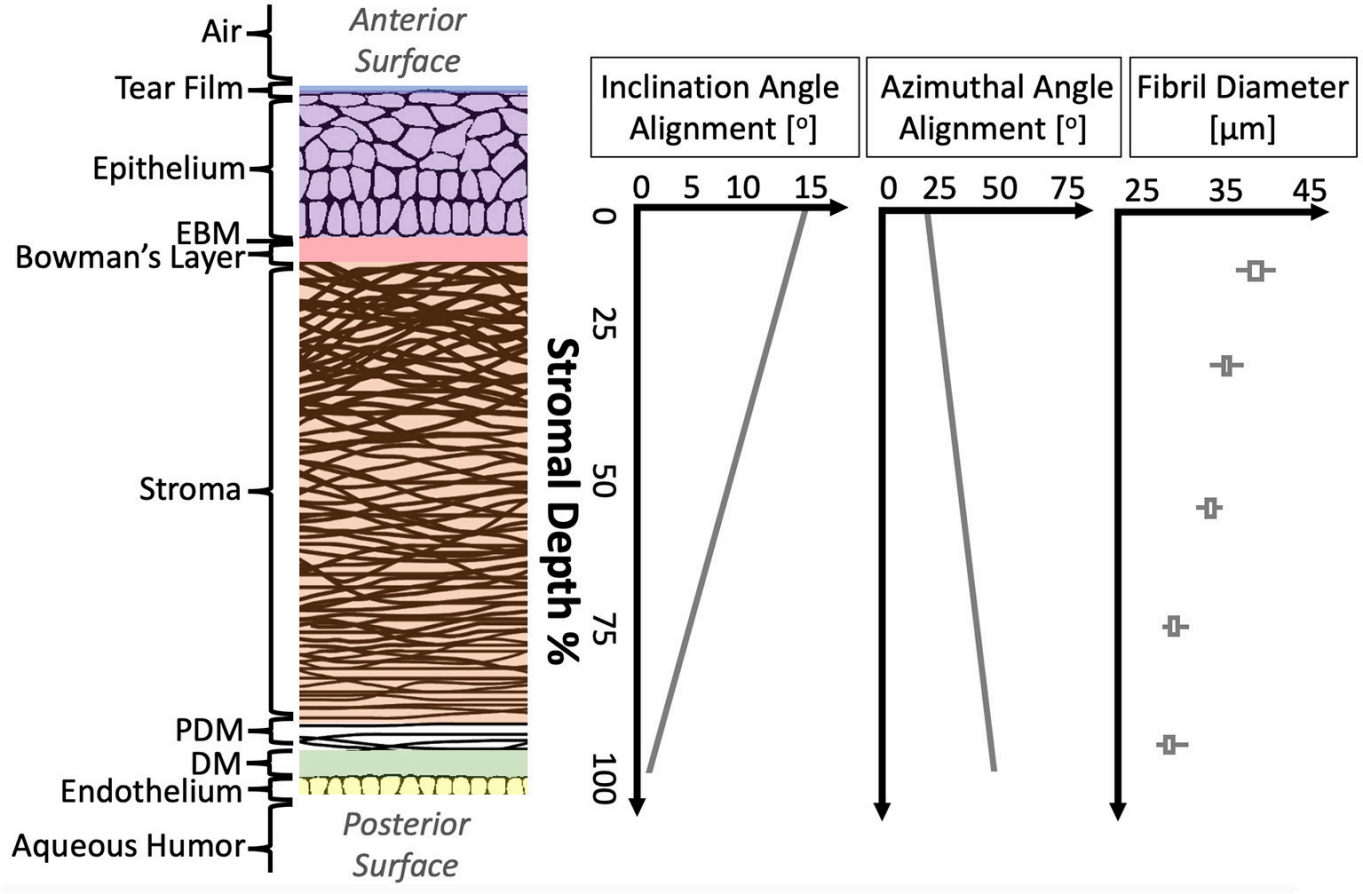
Structural anatomy of human cornea. From left to right: (1) A diagram of human cornea structure. (2) The mean alignment of collagen fibrils with depth in human cornea is shown (Cheng et al., 2015) as well as the mean fibril diameter with depth in porcine cornea (Chang et al., 2018).

As shown in Figure 1 and Table 1, the human cornea is a multi-layered tissue which is ~550 μm thick. Though healthy corneas have over 80% transmission of visible light (400–700 nm) (Boettner and Wolter, 1962), the cornea is dense with proteins, primarily collagen. It is estimated that the residence time of collagen in the cornea is between 2 and 3 years (Smelser et al., 1965). Though the density and structure of the cornea is relatively similar to that of tendon (Marchini et al., 1986), the cornea maintains its transparency through dense, regular packing of proteins within a proteoglycan-rich matrix, allowing light to pass through unimpeded (Knupp et al., 2009).

**Table 1:** Summary of molecular components of human corneal layers.

**Structure**	**General description**	**Constituents**
Tear film	3 μm thick liquid layer which provides smooth optical surface for visual system (Schmoll et al., 2012)	Various lipids, ions (Lam et al., 2014)
Epithelium	50 μm thick layer of cells (Li et al., 1997)	-
Epithelial basement Membrane	0.33 μm thick, mostly collagen and laminin, acellular (Torricelli et al., 2013)	Collagen I (Ben-Zvi et al., 1986), collagen IV (Ben-Zvi et al., 1986), laminin (Millin et al., 1986; Torricelli et al., 2013), fibronectin (Millin et al., 1986), fibrin (Millin et al., 1986), proteoglycans (Torricelli et al., 2013)
Bowman's layer	Measuring around 17 μm thick, is composed of randomly-oriented collagen fibrils (Li et al., 1997; Schmoll et al., 2012).	Similar collagens I and III as stroma (Newsome et al., 1981), more fibronectin than stroma (Newsome et al., 1981), collagen IV and fibronectin present (Tsuchiya et al., 1986)
Stroma	Bulk of cornea, 470 μm, composed of highly organized collagen fibrils bundled into lamellae (Zhou and Stojanovic, 2014).	Lots of collagen I (Newsome et al., 1981), very small amounts of collagen III (Newsome et al., 1981), small amounts of collagen V (Dua et al., 2013), high fibronectin (Newsome et al., 1981) proteoglycan and elastin present
Pre-descemet's membrane (Dua's layer)	15 μm thick layer which, compared to stroma, is tightly packed lamellae and greater space between fibrils (possibly filled with proteoglycans) (Dua et al., 2013)	Same amount of collagen I as stroma (Dua et al., 2013) more collagen IV and VI than stroma (Dua et al., 2013), small amounts of collagen V (Dua et al., 2013), even higher fibronectin than stroma (Newsome et al., 1981), similar proteoglycans as stroma (Dua et al., 2013)
Descemet's membrane	5 μm thick hexagonal lattice of collagen (Sawada et al., 1990; Pavelka and Roth, 2010)	Collagen I (Ben-Zvi et al., 1986), Collagen IV (Newsome et al., 1981; Ben-Zvi et al., 1986), collagen VIII (Tamura et al., 1991)
Endothelium	Single cell layer (Bourne et al., 1997)	-

*Collagen II is not found in the human cornea (Ben-Zvi et al., 1986)*.

Starting from the anterior surface, the tear film is composed lipid, aqueous and proteinaceous layers, ~3 μm thick in total in healthy humans (Azartash et al., 2011). The tear film provides lubrication, protection through soluble immune factors, and a smooth optical surface (Zierhut et al., 2002; Braun, 2012). The 53 μm-thick epithelial layer has no continuous protein networks (Reinstein et al., 2008; Sridhar, 2018). The next layer posteriorly is the epithelial basement membrane (EBM), which is just 0.3 μm thick (Alvarado et al., 1983) and consists primarily of collagen and laminin. They next layer is Bowman's layer. This acellular layer, measuring around 17 μm thick, is composed of randomly-oriented collagen fibrils (Li et al., 1997; Schmoll et al., 2012).

Beneath Bowman's layer lies the corneal stroma, making up the bulk of the corneal tissue. The stroma, ~500 μm thick but variable between individuals (DelMonte and Kim, 2011), is composed of collagen fibrils organized into lamellae which run approximately parallel to the corneal surfaces. At the central cornea, over 200 of these lamellar fibers are stacked on top one another (Bergmanson et al., 2005). Throughout the cornea, these lamellae interweave at varying angles (Cheng et al., 2015). The appropriate spacing between adjacent lamellae and the collagen fibrils within them is critical to corneal transparency and is maintained by proteoglycans. While the stroma is mostly acellular, one keratocyte may be found in every 50,000 cubic microns (Patel et al., 2001).

The Pre-Descemet's membrane (PDM), also called Dua's layer, is largely similar to the adjoining stromal tissues. Differences include a higher density of lamellae and greater spacing between collagen fibrils, indicating differences in proteoglycan distribution. Descemet's membrane (DM), around 3 μm thick, is an acellular fibrous layer secreted by the endothelial cells below. Finally, the endothelium layer is a cell monolayer which has no continuous protein network (Bourne et al., 1997).

Figure 1 and Table 1 provide further details on the molecular constitution of each of these layers. By knowing both the structure and constitution of these layers, some preliminary hypotheses about biomechanics may be drawn. These hypotheses are based on general knowledge of the macromolecular properties of the most common components. Collagens, laminins, fibrins, and fibronectins are known for forming strong fibers and networks, often serving as a sort of scaffolding for tissues (Halper and Kjaer, 2014). Elastin is similar, but, as the name implies, is less brittle than collagen and can tolerate significant strain without permanent deformation. Thus, elastin is often found in tissues which are load-bearing or store mechanical energy (Halper and Kjaer, 2014). Differing subtypes of each of these polymers—collagens, fibrins, elastins, and similar—may point to different mechanical function or different evolutionary/developmental origin (Trelstad R. L., 1973; Exposito et al., 2002). Glycosaminoglycans (GAGs) are highly polar molecules that attract and retain water, often serving to absorb mechanical shocks or otherwise impact viscosity of a tissue. Proteoglycans (PGs) are proteins in which many GAGs attach to one core protein, retaining similar function to the base GAGs (Yanagishita, 1993).

Hypotheses about the mechanical function of each corneal layer can be made based on this information. For instance, because the tear film is a liquid and has no major polymers, it can be assumed to have little direct mechanical contribution to the cornea. Also, the epithelial and endothelial cell layers lack any contiguous protein network, implying that they too will contribute little to the mechanical strength of the cornea. However, the endothelial pump cells are critical to maintaining proper hydration of the cornea, which has a significant effect on corneal mechanics (Fischbarg and Maurice, 2004; Kling and Marcos, 2013; Xia et al., 2014; Shao et al., 2018a; Singh et al., 2018). The stroma, on the other hand, can be assumed to contribute a majority of the mechanical strength to the cornea. On a microstructural scale, the interweaving of collagenous lamellae can be supposed to provide resistance to shear and tensile forces. Because the degree of collagen fibril alignment as well as the diameter of the fibrils vary with depth, it would be straightforward to assume mechanical properties also vary with depth.

**Figure 2:**
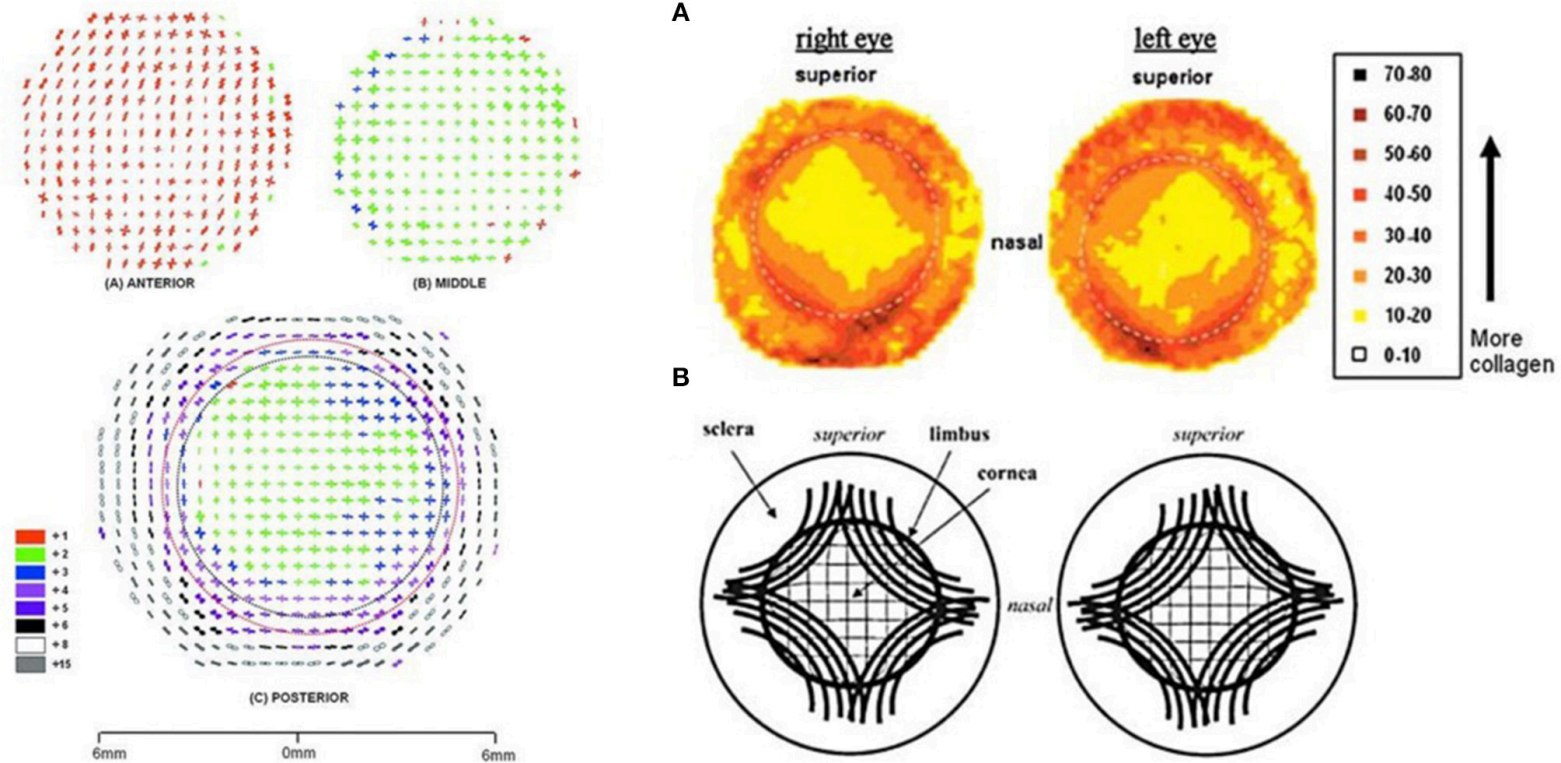
Images of lateral fiber alignment. **Left:** Figure reprinted with permission from reference Abahussin et al. (2009) published by The Association for Research in Vision and Technology (ARVO). Polar plot maps showing collagen fibril orientation at 0.5-mm intervals across varying depth: the anterior third **(A)** and middle 200 μm region **(B)** of a human cornea (P2). The posterior 200 μm of the cornea (with a full thickness scleral rim) **(C)**, is shown after removal of the anterior (red dotted line) and middle (black dotted line) layers from the central 8- to 9-mm region. Polar plots have been scaled down to fit onto the grid as indicated in the color key. In this key, lower numbers (red and green) indicate less fibrillar alignment than higher numbers (white and gray). **Right:** Figure reprinted with permission from reference Boote et al. (2006), published by The ARVO. **(A)** Contour maps of aligned collagen X-ray scatter from a left/right pair of normal human corneas. Scatter from only preferentially aligned collagen fibrils. Dotted circle is the limbus. Scatter listed in arbitrary units. **(B)** Theoretical model of fibrillar arrangement based on **(A)**. Inner solid circle is the limbus.

Finally, there is also considerable lateral heterogeneity in the cornea. Fibril orientation has been found to have a distinct lateral patterning at varying depth layers as shown in Figure 2. This lateral heterogeneity is of interest both because diseases like keratoconus may manifest in heterogeneous ways as a result of tissue structure and because surgical planning may benefit from taking these heterogeneities into account.

## Biomechanical Characterization of the Cornea

While the molecular constitution of the various corneal layers is the foundation of the mechanical properties, it is beyond current technical capabilities to fully calculate mechanical properties directly from constitutive and structural information. Therefore, direct measurements of corneal mechanical properties are made. However, because methods of biomechanical measurement employ a variety of boundary conditions, spatial and temporal scales, and resulting mechanical moduli these methods not necessarily straightforward to interpret or compare. In this section, a summary of methods of measuring corneal biomechanics is presented along with a discussion of their relative strengths and resulting biomechanical measurements.

### Methods of Measuring Mechanical Properties of Corneal Tissues

Before further discussing corneal biomechanics, it is necessary to describe what is encompassed by “mechanical properties.” This broad term applies to almost any measurement of how a sample (the cornea) reacts to some applied force. This force can be large or small and applied over a short or long period of time. Additionally, differing boundary conditions mean that, to varying degrees, a measurement of mechanical properties reflects the tissue of interest or the surrounding tissues and structures.

Mechanical properties which are commonly measured for the cornea include elastic modulus (Young's modulus or storage modulus), shear modulus, and loss modulus (viscosity, also related to stress relaxation, creep, and hysteresis). Each of these properties may be measured by observing the deformation of the sample in response to a specific mechanical loading. Where these moduli differ is in the mathematical model of the material as well as in the duration and direction of the applied force. For instance, the cornea is often considered a viscoelastic tissue, meaning that it has both viscous and elastic characteristics. Both viscous and elastic properties resist deformation, but viscous material resists deformation from applied force over time, while elastic materials more immediately deform with applied force and return to the original state when that force is removed (Glass et al., 2008). Because the cornea exhibits both viscous and elastic characteristics, it is considered viscoelastic. Measuring the mechanical properties of the cornea is further complicated by its heterogeneous and anisotropic structure. Heterogeneous meaning that corneal mechanical properties vary by 3-D spatial location, as in the case of keratoconus (Scarcelli et al., 2014). And anisotropic meaning that an elastic modulus or other property tested along one radial meridian of the cornea will differ from values obtained along different meridians (Elsheikh et al., 2008; Nguyen et al., 2014; Singh et al., 2017).

Taking these complications into consideration, it is important to understand how various methods of mechanical measurement will interact with the cornea. A summary of methods which have been applied to corneal biomechanics can be found in Table 2. While this review will not go into detail describing each method, there are several general considerations to note.

**Table 2:** A summary of biomechanical methods for corneal analysis.

	**Method**	**Spatial regime [mm]**	**Temporal regime [s]**	**Depth mech. resolution [μm]**	**Lateral mech. resolution [μm]**	**Notes**
1	Ocular Response Analyzer (ORA) (Luce, 2005)	2.9	0.5	-	-	*In vivo*, used clinically
2	Corvis ST (Hong et al., 2013)	5	0.1	-	-	*In vivo*, used clinically
3	Inflation testing (Elsheikh et al., 2010)	11	0.5–2.5	-	-	*Ex vivo*
4	Strip Extensiometry (Nash et al., 1982; Richoz et al., 2014; Chang et al., 2018)	6–11	300	-	-	*Ex vivo*
5	Shear Rheometry (Sloan et al., 2014)	3	5	150	-	*Ex vivo*
6	Applanation OCE (Ford et al., 2014)	4	0.1	12	~400	*Ex vivo*
7	Shear wave OCE (Wang and Larin, 2014)	5^*^	0.001	100	~200	*Ex vivo* and *in vivo*
8	Radial Shearing Speckle Pattern Interferometry (Cartwright et al., 2011)	11	3	-	~500	*Ex vivo*
9	Super-sonic Shear Wave Imaging (SSWI) (Tanter et al., 2009)	2	5 × 10^−4^	-	~150	*Ex vivo* and *in vivo*
10	Atomic Force Microscopy (AFM) (Liu et al., 2013; Xia et al., 2014)	5 × 10^−6^	3 × 10^−6^	surface	0.020	*Ex vivo*
11	Brillouin microscopy (Scarcelli et al., 2012)	10^−6^	10^−9^	1	1	*Ex vivo* and *in vivo*
12	Acoustic micro-tapping OCE (Ambrozinski et al., 2016)	10^−4^^*^	0.001	-	~100	*Ex vivo*
13	Acoustic radiation force (Mikula et al., 2014)	0.01	0.002	~100	~1000	*Ex vivo*
14	Phase-Decorrelation OCT (Blackburn et al., 2019)	10^−5^	0.01	40	40	*Ex vivo* and *in vivo*

* *While the diameter of the applied disturbance is very small, the wavelength of the induced elastic wave is the more important consideration. Further, it is assumed that this elastic wavelength will approximately determine the lateral resolution*.

First, some of these methods currently are, or have the potential to be, used *in vivo* because they do not require removal of or significant perturbation to the cornea. However, these methods usually must then disambiguate the mechanical properties of the cornea from contributions of the sclera and the intraocular pressure (IOP). For instance, IOP presses outward on the whole globe, generating compressive (radially) and tensile (circumferentially) forces in the cornea which may be conflated with forces applied for the purpose of measuring elastic properties.

**Figure 3:**
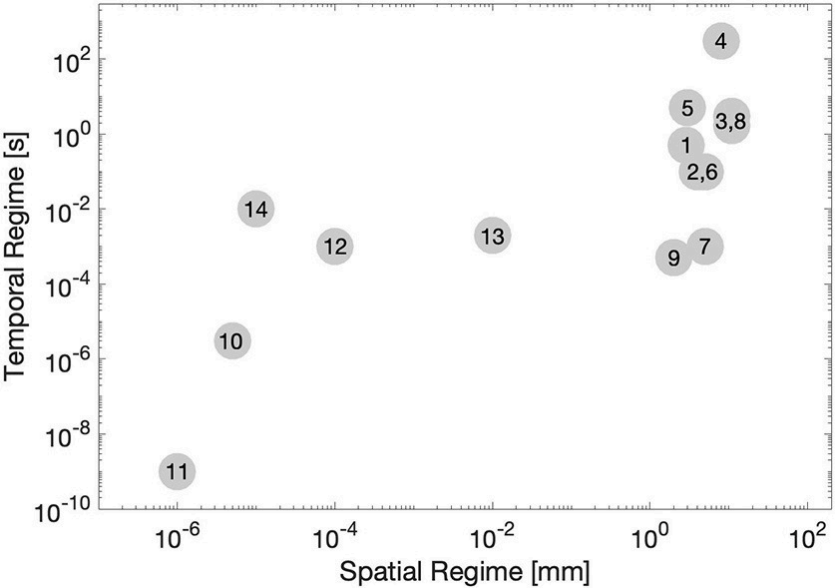
A visual representation of various method of measuring corneal biomechanics, plotted by spatial regime (x-axis, log-scale) and temporal regime (y-axis, log-scale). Numbers correspond to each method listed in Table 2.

Second, these methods cover a wide range of spatial and temporal regimes. In generally, methods which have a shorter temporal scale are expected to more strongly represent elastic properties, while methods with a longer temporal scale can be expected to better represent viscous properties. Also, those methods which operate in a smaller spatial regime may be expected to better approximate material properties of a specific portion of the cornea *in situ*, while methods which operate in a larger spatial regime will almost certainly contain contributions from multiple mechanical structures, even ones outside of the cornea. This is important because sometimes it is desirable to capture mechanical heterogeneity within the cornea, so an appropriate spatial regime must be chosen. Also, in looking at Figure 3, it is easy to appreciate a few trends. One, that the scale of spatial and temporal regime tend to be correlated among methods (that is, there is no method which investigates a very large spatial regime for a very short period of time.

Third, the spatial regime here refers to the area over which a force is applied and the deformation measured. This is distinct from the mechanical resolution of the method, which describes the ability of the method to resolve differences in mechanical properties laterally and in depth. For instance, while compression optical coherence tomography may apply a force over several millimeters of cornea, the way in which internal deformations are tracked means that mechanical heterogeneity may be determined at a much higher resolution (Ford et al., 2014).

Finally, the cornea is a non-linear viscoelastic material. This means that the strain rate and maximum strain will affect the resulting measurement. Additionally, pre-conditioning of the sample may be a significant concern. In summary, it is important to understand the limitations of each method so that an appropriate method may be selected based on the desired results.

### Measuring the Biomechanics of the Cornea

Since at least the 1950's, the mechanical properties of the cornea have been considered and studied (Goldmann and Schmidt, 1957). In the past 60 years, a variety of different measurement methods have been used to investigate the mechanical properties of the cornea, in whole and in part. A summary of the resulting measurements is presented in Table 3. In general, the values reported correspond with expectations based on structural anatomy. The acellular and highly collagenous Bowman's layer, stroma, and Descemet's membrane have higher elastic moduli than the epithelium and endothelium. However, there is also a significant amount of variation in the measurements. Of course, some of this variation is likely due to differences in sample preparation and experimental protocol. However, some of this variation may also be attributed to the different types of mechanical measurement operating in different spatio-temporal tissue regimes. It is important to consider this as a source of variation when comparing techniques.

**Table 3:** Summary of mechanical properties of corneal layers (human tissues unless noted otherwise).

**Structure**	**Measured mechanical property (Method)**
Tear film	Loss modulus (viscosity): 2.33 mPa [Rheometry (Gouveia and Tiffany, 2005)]
Epithelium	Elastic modulus: 0.57 kPa [rabbit, AFM (Thomasy et al., 2014)]
Epithelial basement membrane	Elastic modulus: 7.5 kPa [AFM (Last et al., 2009)], 4.5 kPa [rabbit, AFM (Thomasy et al., 2014)]
Bowman's layer	Elastic modulus: 109.8 kPa [AFM (Last et al., 2012)]
Stroma	Elastic modulus: 33.1 kPa [AFM (Last et al., 2012)], 2 MPa [AFM (Lombardo et al., 2012)], 0.46 MPa [ESPI (Cartwright et al., 2012)], 0.38–1.1 kPa [rabbit, AFM (Thomasy et al., 2014)], 41 kPa [porcine, SW-OCE (Han et al., 2017)] Shear modulus: 10–200 kPa [shear rheometry (Sloan et al., 2014)]
Pre-descemet's membrane (Dua's layer)	No studies available
Descemet's membrane	Elastic modulus: 2.57 MPa [Strip extensiometry (Danielsen, 2004)], 50 kPa [AFM (Last et al., 2009)], 11.7 kPa [rabbit, AFM (Thomasy et al., 2014)]
Endothelium	Elastic modulus: 4.1 kPa [AFM (Thomasy et al., 2014)]

**Figure 4:**
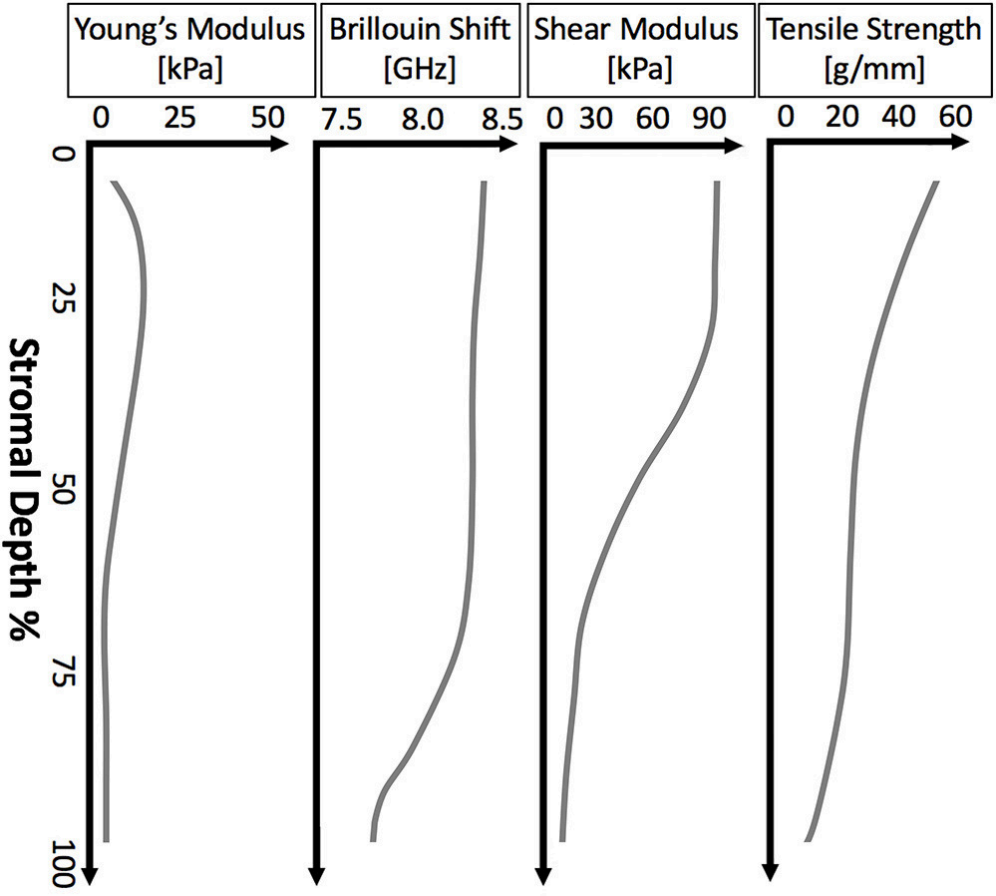
Illustrative plots of depth-dependent corneal properties as measured by various methods. From left to right: the depth-dependent mechanical properties of porcine cornea measured by AFM (Seifert et al., 2014), Brillouin microscopy of human cornea (Scarcelli et al., 2014), shear rheometry of human cornea (Sloan et al., 2014), and extensiometry of human cornea (Randleman et al., 2008b).

While most corneal layers are too thin to allow for depth-dependent measurement, the thick stroma has been shown to have a strong depth-dependent gradient in mechanical properties, even across different measurement methods. The depth-dependent mechanical properties of the cornea from a variety of methods is shown in Figure 4. This gradient of mechanical properties aligns well with our understanding of the collagen fibril structure of the stroma, as highlighted in Figure 1. As expected, regions where the collagen lamellae are more interwoven (the anterior stroma) are consistently found to be stiffer than the posterior cornea (Randleman et al., 2008b; Winkler et al., 2011; Petsche et al., 2012; Scarcelli et al., 2013; Sloan et al., 2014; Thomasy et al., 2014; Wang and Larin, 2014). However, it is interesting to note that this trend hold true for Brillouin microscopy (Scarcelli et al., 2013) and atomic force microscopy (Seifert et al., 2014), techniques for which one might assume lammellar ultrastructure would not have a significant impact because the spatial regime probed by these methods is a much smaller scale than the scale of interweaving lamellae. And in fact, it has been shown that Brillouin microscopy of colloidal gels is not sensitive to microstructure at least on the scale of 10 μm films (Jiménez-Riobóo et al., 1997) and that elastic properties of collagenous tissue measured with atomic force microscopy with a nm-scale tip did not correspond to micrometer-scale mechanical properties and structure (Stolz et al., 2004). Meanwhile, lamellae are around 2 μm thick (Morishige et al., 2011) and the lamellar interweaving which is hypothesized to impart the depth-dependent mechanical variation occurs over tens of microns or more. This might suggest that factors apart from only lammellar interweaving are responsible for the stiffness gradient. Properly accounting for this gradient in computational models of the cornea and in refractive procedures may help to improve patient outcomes.

**Figure 5:**
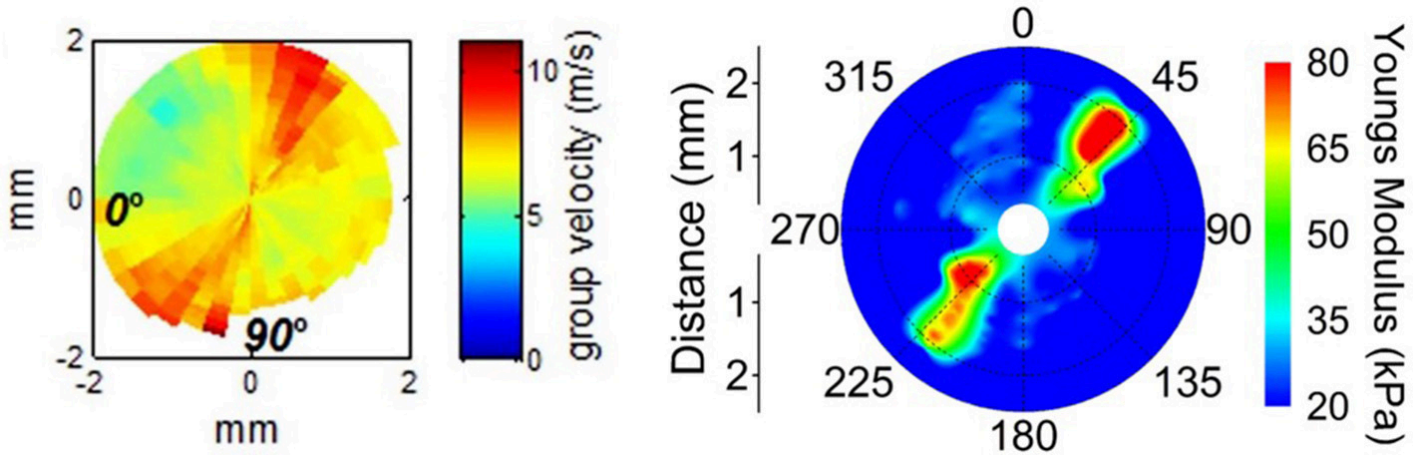
Lateral mechanical anisotropy of the porcine cornea **Left:** Supersonic shear wave imaging of *in vivo* porcine cornea demonstrating corneal anisotropy. Figure reprinted with permission from reference Nguyen et al. (2014), published by The ARVO. **Right:** Shear Wave OCE detection of porcine corneal anisotropy at 20 mmHg intraocular pressure. Figure reprinted with permission from reference Singh et al. (2017), published by The Optical Society (OSA).

Finally, there is the consideration of lateral mechanical heterogeneity arising from the structural heterogeneity described above (see Figure 5). Several studies have found differences in the mechanical properties of mammalian corneas from central to peripheral regions as well as from the nasal-temporal axis to the superior-inferior axis (Elsheikh et al., 2008; Ford et al., 2014; Nguyen et al., 2014; Mikula et al., 2016; Singh et al., 2016). Other studies have found a distinct radial axis along which a whole-cornea anisotropy revolves (Nguyen et al., 2014; Singh et al., 2017). This axis is assumed to be related to a similar axis observed in the collagen fiber alignment, and which may vary by up to 13° from person to person (Bone and Draper, 2007). Characterizing this sort of anisotropy, both in the average patient population as well as for individual patients would allow for treatments to work in harmony with the existing tissue properties to improve outcomes.

### Modeling the Mechanics of the Normal Cornea

Since the late 1980's (Hanna et al., 1989; Buzard, 1992), finite element modeling (FEM) has been used to simulate the mechanical response of the cornea to refractive surgery. These early models recognized that corneal tissue is anisotropic due to its lamellar organization, even before there were any studies which quantified the mechanical nature of the anisotropy of the cornea. Despite many simplifying assumptions, these models provided a useful foundation for surgical planning and demonstrated how mechanical modeling could explain the refractive effects achieved by modulating the length and depth of surgical incisions. More recently, models have become more nuanced by taking into consideration patient-specific geometry (Roy and Dupps, 2011; Studer et al., 2013; Sánchez et al., 2014) as well as modeling based on the alignment and distribution of collagen fibrils themselves (Pandolfi and Holzapfel, 2008). The clinical predictive value of such models was demonstrated by the validation of patient-specific models against real LASIK outcomes (Seven et al., 2016).

## Age-related Change in Mechanics

Many tissues in the human body change with age, typically becoming less flexible (Geraghty et al., 2015). The cornea, too, has been demonstrated to change in structure, composition, and mechanical properties with age. Understanding these changes may be important for optimizing diagnostic procedures and surgical algorithms to optimize patient outcomes.

### Changes in Structure and Composition Associated With Aging

With aging come both composition and structural changes in the cornea. Some collagens change level of expression. Table 4 provides a summary of structural and compositional changes which have been noted with age. Other recent reviews have provided a more in depth explanation of age-related cellular and molecular corneal changes (Cerulli and Missiroli, 2008).

**Table 4:** Summary of corneal microstructural changes associated with age.

**Structure**	**Change with age**
Whole cornea	• Increase in non-enzymatic crosslinking (Malik et al., 1992) • Decrease in GAGs (Pacella et al., 2015) • Decrease in hydration stability (Polse et al., 1989) • Increase in collagen glycation (Malik et al., 1992) • Decrease in nerve density (Gipson, 2013) • No change in CCT (Cerulli and Missiroli, 2008) • Increase in densitometry (Garzón et al., 2017) • Increase in reactive oxygen species (Nita and Grzybowski, 2016)
Tear film	No significant effects found (Craig and Tomlinson, 1998)
Epithelium	• Decrease in cell density (Zheng et al., 2016) • Cell diameter increases (Gambato et al., 2015)
Epithelial basement membrane	Thickening (Alvarado et al., 1983; Gipson, 2013)
Bowman's Layer	No noticeable change in collagens (Newsome et al., 1981), but disruption of structure (Tsuchiya et al., 1986)
Stroma	• Collagen III decreases throughout neonatal development (Ben-Zvi et al., 1986) • Increase in refractive index (Patel et al., 2011; Amparo et al., 2012) • Increase in collagen fibril diameter (Daxer et al., 1998) • Increase in number of collagen molecules (Daxer et al., 1998) • Increase in intermolecular Bragg spacing (Daxer et al., 1998) • Decrease in interfibrillar spacing (Kanai and Kaufman, 1973) • Decrease in keratocyte density (Berlau et al., 2002; Niederer et al., 2007; Gipson, 2013; Gambato et al., 2015; Zheng et al., 2016)
Pre-descemet's membrane (Dua's layer)	No known changes
Descemet's membrane	• Thickening over lifetime (Gipson, 2013) • Collagen IV increase after birth (Ben-Zvi et al., 1986) • Fibronectin increase after birth (Ben-Zvi et al., 1986)
Endothelium	Decrease in cell density (Bourne et al., 1997; Niederer et al., 2007; Gipson, 2013; Gambato et al., 2015; Zheng et al., 2016)

Of particular interest is the age-related increase of non-enzymatic crosslinks in the stroma. Not only does this provide the most direct explanation for widely-observed age-related increase in stiffness, but it also suggested that corneal crosslinking (CXL, discussed in section Cornea Crosslinking-Related Change in Mechanics) may be biomechanically similar to age-related crosslinking.

### Changes in Mechanical Properties Associated With Aging

As a result of the structural and compositional age-changes, the mechanical properties of the cornea change as well. Table 5 provides a summary of methods which have detected age-associated mechanical changes in the cornea along with the coefficient of determination (*r*^2^ value) relating the change to age. The coefficient of determination is a normalized measure of how well a given model matches observations. In the case of age-related cornea studies, the model is often a simple linear regression of measured values against patient age, however some models use a higher-order or multivariate fits. In general, the cornea is found to increase in stiffness roughly linearly with age while decreasing in viscous behavior (such as hysteresis and creep) with age. While there are a relatively small number of studies on the mechanics of human corneal aging, an intriguing parallel may be drawn with corneal crosslinking (CXL) treatment (section Cornea Crosslinking-Related Change in Mechanics). Like aging, the primary cause for increase in stiffness in CXL is the addition of non-enzymatic crosslinks. Further, the typical stabilization of keratoconus (section Keratoconus-Related Change in Mechanics) after a certain age may be related to the naturally-occurring increase in non-enzymatic crosslinks which mimics CXL.

**Table 5:** Summary of corneal biomechanical changes associated with age.

**Structure**	**Trend (*r*^**2**^ with age, method)**
Whole cornea or globe	• Ocular Rigidity increases [0.073, pressure-volume measurements (Pallikaris et al., 2005)] • Stiffness increases [0.2866, inflation testing (Elsheikh et al., 2010)] • Corneal hysteresis decreases [0.0289, ORA (Kamiya et al., 2009)] • Corneal resistance factor decreases [0.0324, ORA (Kamiya et al., 2009)] • SP-A1 stiffness parameter increases [Corvis ST (Roy and Dupps, 2011)] • Relaxation rate increases [murine, Shear Wave OCE (Li et al., 2014)]
Tear film	Reduction in tear film stability [0.25–0.29, fluorescein break up (Patel and Farrell, 1989)]
Epithelium	No mechanical studies
Epithelial basement membrane	No significant increase in elastic modulus with age [AFM (Last et al., 2012)]
Bowman's layer	No significant increase in elastic modulus with age [AFM (Last et al., 2012)]
Stroma	• No significant increase in elastic modulus with age [AFM (Last et al., 2012)] • Increase in elastic modulus with age [AFM (Xia et al., 2014)] • Inter-lamellar cohesive strength increases [0.45, Extensiometry (Randleman et al., 2008b)]
Pre-descemet's membrane (Dua's layer)	No mechanical studies
Descemet's membrane	Small increase in elastic modulus with age [AFM (Last et al., 2012)]
Endothelium	No mechanical studies

The relationship between age, non-enzymatic crosslinks, and corneal biomechanics becomes even more interesting when keratoconus (discussed further in the next section) is considered. Keratoconic corneas are known to have an upregulation of matrix metalloproteinases (MMPs) along with other pro-collagen and proteolytic enzymes (Shetty et al., 2015). Age, however, is inversely correlated with risk of keratoconus (Ertan and Muftuoglu, 2008), further progression of keratoconus (Li et al., 2007), and risk of post-LASIK ectasia (Randleman et al., 2008a), all of which suggest that age-related crosslinks might be protective against keratoconus and other ectasias. This hypothesis is further supported by studies showing that CXL treatment directly increases resistance to enzymatic digestions (Spoerl et al., 2004).

Another related but independent cause of non-enzymatic crosslinks is diabetes (Sady et al., 1995), which itself is related to age (Wild et al., 2004). In diabetic patients, excess sugars, advanced Maillard reaction, and lysyl oxidase mediated crosslinking occur in the cornea (Sady et al., 1995) and cause the cornea to become stiffer (Goldich et al., 2009; Sahin et al., 2009; Kotecha et al., 2010; Scheler et al., 2012). Likely, this increase in non-enzymatic crosslinks and mechanical stiffness is the cause for the reduced likelihood of severe keratoconus in diabetic patients (Kuo et al., 2006).

### Modeling Mechanics of Aging and Significance to Clinical Practice

As patients age, there are a number of associated clinical changes in the morphology and mechanics of the cornea, in addition to changes summarized in Tables 4, 5. For instance, there is a significant shift from proportion of “with the rule” (vertical meridian steeper) astigmatism to “against the rule” (horizontal meridian steeper) astimatism (Salvi et al., 2006). From birth to adulthood, there is a general flattening of the cornea (Gordon and Donzis, 1985).

Because of the dependence of corneal stiffness on age, age is an important consideration in whole-eye diagnostics and treatment involving the cornea. For instance, it has been determined that age must be considered and corrected for in Goldmann applanation tonometry in order to get precise intraocular pressure readings (Tonnu et al., 2005; Elsheikh et al., 2011, 2013) Further, as stiffness was modulated in a finite element model of the cornea to represent the normal range of properties encountered in human tissue studies, it was found to be an important factor in *laser-assisted in situ keratomileusis* (LASIK) responses. In comparisons of stiffer and weaker corneas in otherwise identical whole-eye models, the same simulated LASIK procedure for myopia was found to under-correct myopia (inadequately flatten) in weaker corneas and overcorrect (excessively flatten) in stiffer corneas due to the response of the remaining corneal tissue (Roy and Dupps, 2009).

Based on this analysis, it can be inferred that because older patients tend to have stiffer corneas, LASIK procedures would tend toward over-correction in older patients and under-correction in younger patients. This is indeed the case clinically. Because under-correction often leads to enhancement surgeries, which themselves are associated with elevated ectasia risk (Randleman et al., 2017), there is clinical interest in improving LASIK planning algorithms to incorporate age or corneal biomechanics. Including these factors may improve patient visual outcomes in addition to lowering risks associated with the procedure.

### Keratoconus-Related Change in Mechanics

Ectasias, specifically keratoconus, are potentially debilitating ocular diseases with a significant biomechanical component. For over 30 years, it has been known that keratoconus is related to an explicit biomechanical weakening of the cornea, as measured by strip extensiometry (Andreassen et al., 1980). Additionally, a host of molecular and structural changes have been found to be related to progression of the disease. However, the full etiology of keratoconus and other ectasias is not completely understood. And even though significant advances in understanding of keratoconus have occurred in the past 40 years, keratoconus is still primarily diagnosed by progressively worsening visual acuity and distortions in corneal 3D anterior surface topography or 3D tomography.

The introduction of corneal collagen crosslinking (discussed in section Cornea Crosslinking-Related Change in Mechanics) as treatment for early- and mid-stage keratoconus has made it particularly imperative to diagnose the disease early so patients' vision may be preserved without corneal transplants. Because it is hypothesized that biomechanical changes may precede topographic changes (Roy et al., 2013), a thorough understanding of biomechanics may aid in diagnosis of early-stage keratoconus. In this section, keratoconus-associated changes in structure and composition will be summarized, followed by biomechanical observations and relation to clinical practice.

### Changes in Structure and Constitution Associated With Keratoconus

Even before keratoconus was properly identified in the medical literature, changes in corneal structure due to keratoconus were noted (Grzybowski and McGhee, 2013). Indeed, unlike many diseases, the direct cause of the patients' complaint—reduced visual acuity—is plain to see in the conical distortion of the cornea. While medical science has become much more adept at characterizing keratoconus, identifying a single trigger which causes the disease to manifest has remained elusive. Today, the most plausible explanation is that keratoconus is a multifactorial disease of genetics and environment (Gordon-Shaag et al., 2015). While the exact causes are still unknown, a large number of structural and molecular markers have been found to be associated with keratoconus. There has been significant variation between studies of the microstructural properties of keratoconus, in part because some studies focused on tissue in the cone regions, while other studies considered the whole cornea. A summary of some of these structural indicators is provided in Table 6. While no single structural or compositional change fully explains the conical distortion in keratoconus, the increase in enzyme activity would seem to have the most direct link to the change in biomechanical properties, as discussed in the next section.

**Table 6:** Summary of corneal microstructural changes associated with keratoconus.

**Structure**	**Change with KCN**
Whole cornea	• Changes in expression of laminin (Tuori et al., 1997), collagen VII (Tuori et al., 1997) • Increased enzyme activity (Shetty et al., 2015)
Tear film	Various biomarkers (Lema et al., 2010; Balasubramanian et al., 2012)
Epithelium	• Thinning (Roy et al., 2013) • Apoptotic cells (Sherwin and Brookes, 2004) • Focal thinning over stromal cone region and thickening peripherally (Reinstein et al., 2009)
Epithelial basement membrane	• Increase in collagen (1/2) IV (Tuori et al., 1997) • Decrease in collagen (5/6) IV (Tuori et al., 1997) • Changes in fibronectin (Kenney et al., 1997) • Changes in collagen (3/5) IV (Kenney et al., 1997) • Changes in laminin (Kenney et al., 1997) • Decrease in fibrin (Millin et al., 1986)
Bowman's layer	• Discontinuities and defects (Scroggs and Proia, 1992; Tuori et al., 1997) • Fibrotic regions where discontinuous (Kenney et al., 1997) • Deposits of collagen VIII (Kenney et al., 1997), fribillin (Kenney et al., 1997), collagen (1/2) V (Kenney et al., 1997)
Stroma	• No noticeable change in collagens present (Newsome et al., 1981; Tsuchiya et al., 1986) • Significant change in lamellar organization (Meek et al., 2005) • Changes in lateral fiber alignment (Akhtar et al., 2008) • Thinning (Scroggs and Proia, 1992; Tuori et al., 1997) • Increased fibril density (Akhtar et al., 2008) • Decreased fibril diameter (Akhtar et al., 2008) • Increased proteoglycan density (Akhtar et al., 2008) • Fewer keratocytes (Sherwin and Brookes, 2004)
Pre-descemet's membrane (Dua's Layer)	No known changes
Descemet's membrane	• No immunohistochemcial change (Kenney et al., 1997) • Ruptures, folds (Sherwin and Brookes, 2004)
Endothelium	Apoptotic cells (Sherwin and Brookes, 2004)

### Changes in Mechanical Properties Associated With Keratoconus

Given the structural changes associated with keratoconus, it is expected that mechanical changes will also take place. So while mechanical changes have long been associated with keratoconus, the relative lack of adequate measurement techniques, particularly *in vivo* techniques, has stymied further study. Within the last decade, however, there have been significant advances in both *in vivo* and *ex vivo* corneal biomechanical measurement methods. The motivation for this is several-fold. First, increasing our understanding of the relationship between corneal structure and function in disease will offer insight into which factors most contribute to pathologic phenotypes. Second, there is reason to believe (Yenerel et al., 2010) that early-stage pathological mechanics may be possible to detect earlier than early-stage pathologic structure (Vinciguerra et al., 2017). The long-sought “gold standard” would be an *in vivo* method which could reliably detect biomechanical changes in the cornea prior to pathologic topography.

In general, studies have found weakening of the entire cornea, as well as focal weakening in the lateral location of the keratoconus “cone.” Table 7 summarizes the results of a variety of methods which have been used to study the biomechanics of keratoconus. For each method, the *t*-value was calculated from the reported changes in the mechanical coefficient. The *t*-value is a statistical coefficient which measures the significance between population means given their standard deviation. The authors of this review calculated *t*-values for studies which give the sample sizes, mean value of a normal and keratoconus group (or groups, if both forme fruste and manifest keratoconus were investigated), and standard deviation of each. In general, a high *t*-value means that method clearly distinguished keratoconus from normal corneas. Of course, there are many factors which affect this *t*-value apart from method sensitivity, including enrollment criteria and sample sizes for each study. However, there is still value in comparing relative *t*-values to contextualize measurement methods.

**Table 7:** *T*-values of studies which seek to detect keratoconus based on biomechanics or structure.

**Coefficient, method**	**Ref**	**Normal**			**Forme fruste keratoconus**				**Manifest keratoconus**			
		***N***	**Mean**	**STD**	***N***	**Mean**	**STD**	***t*-value**	***N***	**Mean**	**STD**	***t*-value**
Corneal hysteresis, ORA	Yenerel et al., 2010	63	11.43	1.52	34	9.21	1.38	8.73	36	9.21	1.38	12.67
	Viswanathan et al., 2015	50	10.07	1.73	-	-	-	-	100	8.08	1.77	8.70
	Johnson et al., 2011	115	11	1.4	42	8.8	1.42	10.25	73	7.9	1.3	17.9
	Shah et al., 2007	207	10.7	2	-	-	-	-	93	9.6	2.2	6.03
Corneal resistance factor, ORA	Yenerel et al., 2010	63	11.53	1.83	34	8.21	1.64	11.94	36	6.79	1.81	16.82
	Johnson et al., 2011	115	11.1	1.6	42	8.6	1.3	11.8	73	7.3	1.4	20.89
	Viswanathan et al., 2015	50	9.82	1.88	-	-	-	-	100	6.87	2.04	12.25
Brillouin shift, Brillouin microscopy	Shao et al., 2018b	47	5.721	0.024	-	-	-	-	8	5.67	0.03	0.78
Stiffening constant, Strip extensiometry	Nash et al., 1982	9	59	3.47	-	-	-	-	6	45.35	3.82	13.62
**FOR COMPARISON, EXAMPLES OF STRUCTURAL-ONLY METHODS**												
Central corneal thickness (Pachymetry)	Ambrósio et al., 2011	113	550.5	33.8	-	-	-	-	44	483.3	41.8	60.07
	Saad and Gatinel, 2010	72	554.6	36.1	40	524.3	37.0	25.37	31	487.5	52.1	45.42
Posterior elevation thinnest pachymetry	Saad and Gatinel, 2010	72	19.7	8.6	40	26.3	11.0	10.5	31	73.2	37.5	46.4
Ambrósio's relational thickness-Ave	Ambrósio et al., 2011	113	696.2	462.2	-	-	-	-	44	251	119	170.57

### Modeling Mechanics of Keratoconus and Significance to Clinical Practice

While the complete etiology of keratoconus is not understood, the mechanical effects have been simulated in finite element modeling and shown to agree with observed disease states (Roy and Dupps, 2011). Pandolfi and Manganiello were able to simulate a keratoconus phenotype by reducing the stiffness of the cornea (Pandolfi and Manganiello, 2006). Carvalho et al. (2009) also found that by reducing stiffness locally, keratoconus-like cones formed. Roy and Dupps (2011) demonstrated that by incrementally simulating a focal weakness in a finite element model of the cornea, the cornea incrementally took the cone shape characteristic of keratoconus. The fact that focal weakening alone, independently of other factors, is predicted to cause a cone shape suggests that focal weakening might be the common, final pathway to the multifactorial pathogenesis of keratoconus. Additionally, the further development of corneal biomechanical measurement technologies provides hope that there may soon be fast and reliable methods to screen for early keratoconus.

## Cornea Crosslinking-Related Change in Mechanics

Since its introduction in 2003(Wollensak et al., 2003), corneal crosslinking (CXL) has become an established practice in the care of keratoconus patients (Meiri et al., 2016). This method, in the most popular “Dresden” protocol, applies a dilute riboflavin solution to the de-epithelialized surface of the cornea for 30 min, followed by 30 min of ultraviolet radiation (Wollensak et al., 2003). This treatment photochemically (Kamaev et al., 2012; Richoz et al., 2013; Semchishen et al., 2015) induces additional crosslinks between proteoglycans and collagens in the cornea stroma, stiffening the cornea and making it less susceptible to enzymatic digestion (Hayes et al., 2013). This stiffening usually stops the progression of keratoconus and can even provide a mild correction to the keratoconic cone (Meiri et al., 2016).

### Changes in Structure Associated With CXL

Because CXL is an exogenous treatment, there are relatively few compsitional changes directly associated with treatment. *In vitro*, CXL treatment on human corneal tissue samples was associated with increases in the density, diameter, and area of corneal fibers, as well as corneal thickness (Choi et al., 2013). *In vitro* porcine corneas similarly showed altered collagen architecture, an increase in fibril diameter but a decrease in fibril density (Chang et al., 2018). *In vivo* rabbit cornea also showed an increase in collagen fibril diameter 4 h after CXL treatment (Wollensak et al., 2004). However, some of these effects may be accounted for by the edema or other factors transiently induced by CXL treatment (Chang et al., 2018).

Other structural changes have been observed in humans in the months following a CXL treatment. In one study of human corneal tissue treated *in vivo*, it was found that 6 months after CXL treatment there was an increase in the diameter and inter-fibrillar spacing of stromal collagen fibrils, while a reduction in the proteoglycan area (Akhtar et al., 2013).

### Changes in Mechanics Associated With CXL

To summarize the numerous biomechanical measurement methods Table 8 is provided. For all methods, studies were completed on human tissue using the Dresden protocol unless otherwise noted. For each method, the *t*-value was calculated from the reported changes in the mechanical coefficient. The *t*-value is a statistical coefficient which measures the significance between population means given their standard deviation. The authors of this review calculated *t*-values for studies which give the sample sizes, mean value of a normal and keratoconus group, and standard deviation of each.

**Table 8:** *T*-values of studies which analyzed the mechanics of corneas before and after crosslinking treatment.

**Parameter, method**	**References**	**No CXL treatment**			**Post-CXL treatment**			**Test statistic (*t*-value)**
		***N***	**Mean**	**STD**	***N***	**Mean**	**STD**	
Corneal hysteresis, ocular response analyzer	Viswanathan et al., 2015	100	8.08	1.77	25	8.56	1.68	1.65
Corneal resistance factor, ocular response analyzer	Viswanathan et al., 2015	100	6.87	2.04	25	7.47	1.88	1.94
Elastic modulus, shear wave OCE	Han et al., 2017	4	41.8	8.1	4	87.3	9.5	21.7
Viscous modulus, shear wave OCE	Han et al., 2017	4	0.7	0.2	4	0.1	0.1	2.19
% Change elastic modulus, brillouin microscopy	Webb et al., 2017	-	-	-	4	5.2	0.2	16.44
Brillouin shift, brillouin microscopy	Shao et al., 2018b	8	5.697	0.029	16	5.725	0.03	0.377
Strain, strip extensiometry	Richoz et al., 2014	5	0.26	0.01	5	0.12	0.03	1.57
Tangent modulus, strip extensiometry,	Chang et al., 2018	6	1.38	0.17	6	2.09	0.17	2.98
Elastic modulus, atomic force microscopy (porcine)	Seifert et al., 2014	8	8.2	1.7	4	46	17	17.89
	Matteoli et al., 2016	10	0.6	0.58	10	1.58	1.04	2.43
Elastic modulus, inflation (porcine)	Blackburn et al., 2019	15	0.692	0.3	23	1.096	0.3	2.22
	Matteoli et al., 2016	12	2727	238	12	3868	502	145.3
Elastic modulus, radial shearing speckle pattern	Cartwright et al., 2012	3	0.46	0.2	3	2.06	0.22	4.27
Decorrelation % change, phase decorrelation OCT	Blackburn et al., 2019	5	41.55	9.64	5	2.83	12.56	18.38
Elastic modulus, supersonic shear imaging	Tanter et al., 2009	4	190	32	4	890	250	83.37
% Change elastic modulus, supersonic shear imaging	Nguyen et al., 2012	-	-	-	4	56	15	20.44

In general, a high *t*-value means that method clearly distinguished corneas treated with crosslinking from those that were untreated or given a sham treatment. Viewing methods in this way may be of assistance to researchers hoping, for instance, to assess novel crosslinking techniques with the most sensitive mechanical measurement method.

### Modeling Mechanics of Cornea Crosslinking and Significance to Clinical Practice

Finite element modeling simulations (Roy and Dupps, 2011) suggest that the clinically-observed flattening of the keratoconus cone post-CXL treatment is a direct result of the CXL-mediated stiffening. Further simulations suggest that specific crosslinking patterns could optimize the corneal flattening and improve patient's visual acuity. This patterning could be used not just to reduce the keratoconic cone, but perhaps to non-surgically reduce astigmatism in non-keratoconus patients with fewer aberrations than traditional relaxing incisions (Seven et al., 2014). This work has also lead to clinical studies to determine the effectiveness of patterned CXL in correcting myopia and hyperopia (Elling et al., 2018).

## Hormone-Related Changes in Corneal Mechanics

In addition to aging, ectatic disease, and crosslinking, hormones have been shown to affect corneal biomechanics. Tabibian et al. (2017) showed that pregnancy-related changes in thyroid hormones are associated with changes in corneal hysteresis and corneal resistance. Several studies have highlighted patients, some of whom previouslyH received CXL treatment for keratoconus, having rapid progression of keratoconus after significant hormone changes (Yuksel et al., 2016; Lee et al., 2018; Torres-Netto et al., 2019). Further, cornea biomechanics are shown to change with the menstrual cycle (Goldich et al., 2011). However, studies have generally not found differences in cornea biomechanics between men and women. The effects of hormones on cornea biomechanics is an emerging field of study which will require further investigations to determine clinical relevance.

## Conclusion

While the biomechanical behavior of the cornea in inexorably linked to its visual function, the complexity of the cornea has historically made mechanical analysis challenging. A wide variety of techniques, both *ex vivo* and *in vivo* have been used in recent years to better understand the relationship between physiologic states, corneal biomechanics, and visual function. However, this variety of techniques encompasses a very wide range of spatial and temporal regimes, making it difficult, if not impossible, to compare results between studies. This challenge is illustrated by the many orders of magnitude difference in elastic modulus of the corneal stroma, as reported among various studies. This variation is not the fault of any particular study, but an expected outcome when interrogating a mechanically complex structure on scales that range from nanometers to centimeters and milliseconds to minutes.

Despite this, studies of corneal biomechanics have produced results which both conform to theoretical expectations and illuminate new understandings of disease and treatment. For instance, corneal biomechanics studies have explained age-related overcorrection of patients' vision due to refractive surgery (Waring et al., 1987; Akura et al., 2000; Roudakova et al., 2000; Roy and Dupps, 2009). Additionally, biomechanical studies have confirmed the theory of focal weakening in keratoconus (Roy and Dupps, 2011; Shao et al., 2018b). Further biomechanics studies have been used to assess the efficacy of various corneal crosslinking treatments. Looking forward, the field can expect the maturation of corneal biomechanical assessments and further advances in personalized biomechanical modeling, both of which will serve to improve the treatment and well-being of patients.

## Author Contributions

BB contributed to literature researches, the preparation of graphics and tables, analysis of literature, and preparation the manuscript. MJ contributed to interpretation of literature and data and its form of presentation in the work. AR contributed literature analysis, critical feedback, and revision work to the manuscript. WD contributed to the conception of the paper, the literature research, critical feedback and revisions, as well as interpretation of data and literature.

### Conflict of Interest Statement

Intellectual property related to cornea biomechanics held at Case Western Reserve University (BB, MJ, AR, and WD) and Cleveland Clinic (WD). WD is a consultant for Avedro Inc. and Alcon.
